# Towards clinical integration of real-time 3D coronary stent reconstruction using contrast-free rotational angiography in percutaneous coronary intervention

**DOI:** 10.1093/ehjimp/qyaf085

**Published:** 2025-06-20

**Authors:** Ioannis Skalidis, Ioannis Kachrimanidis, Leonidas Koliastasis, Michalis Hamilos, Emmanouil Skalidis

**Affiliations:** Institut Cardiovasculaire Paris-Sud, Hôpital Jacques Cartier, Ramsay-Santé, 6 Avenue du Noyer Lambert, Massy 91300, France; School of Medicine, University of Crete, Heraklion, Greece; 1st Department of Cardiology, Hippokration General Hospital, National and Kapodistrian University of Athens, Athens 11527, Greece; 1st Department of Cardiology, Hippokration General Hospital, National and Kapodistrian University of Athens, Athens 11527, Greece; Cardiology Department, University Hospital of Heraklion, Heraklion, Crete, Greece; Cardiology Department, University Hospital of Heraklion, Heraklion, Crete, Greece

Accurate assessment of coronary stent expansion is essential for optimal percutaneous coronary intervention (PCI) outcomes, yet current imaging approaches present trade-offs between diagnostic precision, invasiveness, and workflow integration. The study by Benamer *et al*.^1^ introduces and evaluates an innovative imaging approach—C-arm Motion Compensated Computed Tomography (CMCT)—for *in situ* 3D coronary stent reconstruction during PCI. Using standard angiographic equipment without the need for contrast agents or intravascular imaging tools, this proof-of-concept, first-in-human study addresses an important unmet need: real-time, volumetric assessment of stent deployment directly in the cath lab.

The results are promising. In over 70% of cases, 3D reconstructions were rated as ‘excellent’ or ‘good’ by all three independent reviewers, with strong inter-observer agreement. The correlation between image quality and patient body mass index highlights the relevance of patient-specific factors and may inform further optimization. The technique’s contrast-free, low-radiation profile enhances its potential as a safe and accessible tool, especially in centres with limited access to intravascular imaging.

A particularly notable feature of the method is the use of angioplasty balloon markers to compensate for cardiac motion—an elegant adaptation of Cone Beam Computed Tomography, for a dynamic, intravascular setting. However, this motion correction strategy has primarily been applied to straight or mildly curved vessels. Its robustness in more complex anatomies—such as tortuous segments or bifurcations—remains to be fully assessed. How stent length, overlap, or vessel curvature may affect marker tracking and reconstruction fidelity is a valuable area for further investigation, particularly as these scenarios often present the greatest need for advanced imaging.

The study also includes a limited yet encouraging comparison with optical frequency domain imaging, showing a mean discrepancy of just 3.2% in stent area measurements. While this suggests potential for quantitative accuracy, broader validation against optical coherence tomography (OCT) and intravascular ultrasound (IVUS) across varied lesion types and stent platforms will be necessary to establish clinical equivalence and support integration into procedural decision-making.

Another intriguing aspect is the comparison with StentViz, a widely used 2D enhancement tool. In the subset of cases with both modalities, CMCT was consistently preferred by reviewers, reinforcing the value of volumetric imaging. However, StentViz remains a rapid and efficient tool in routine practice. Clarifying how CMCT might complement or selectively enhance such 2D tools—particularly in ambiguous or high-risk lesions—could help define practical implementation pathways.

While this study focused on offline analysis, future work should explore real-time reconstruction and interpretation during live procedures. Key aspects such as reproducibility across operators, learning curve, and sensitivity to physiological variability (e.g. arrhythmias or breath-holding inconsistencies) will influence clinical adoption and scalability.

In summary, Benamer *et al*. deliver a technically robust and clinically relevant first-in-human study that expands the potential of angiographic systems for intraprocedural 3D stent imaging. As a contrast-free, device-independent modality, CMCT may serve as a pragmatic adjunct in PCI—particularly in settings where intravascular imaging is limited. Further prospective studies will be essential to define its diagnostic value, clinical impact, and long-term role in routine practice.

## Data Availability

No new data were generated or analysed in support of this research.

## Lead author biography

Ioannis Skalidis, MD, PhD, is an interventional cardiologist at Institut Cardiovasculaire Paris Sud (ICPS) in France. He completed his core cardiology training at the University Hospital of Lausanne (CHUV) in Switzerland and holds a PhD degree from the University of Crete. Dr Skalidis is a Swiss Board (FMH) certified cardiologist and serves as a member of the European Society of Cardiology’s e-Cardiology Working Group. His academic interests encompass coronary physiology, TAVR, and the integration of artificial intelligence and simulations in transcatheter coronary and structural heart interventions. He has authored over 80 peer-reviewed publications.
